# Genetic and genomic approaches for R-gene mediated disease resistance in tomato: retrospects and prospects

**DOI:** 10.1007/s00299-012-1234-z

**Published:** 2012-02-18

**Authors:** M. R. Ercolano, W. Sanseverino, P. Carli, F. Ferriello, L. Frusciante

**Affiliations:** Department of Soil, Plant, Environmental and Animal Production Sciences, University of Naples ‘Federico II’, Via Università 100, 80055 Portici, Italy

**Keywords:** *Solanum lycopersicum*, Disease resistance, Genomic tools, Emerging technologies, New breeding methods

## Abstract

Tomato (*Solanum lycopersicum*) is one of the world’s most important vegetable crops. Managing the health of this crop can be particularly challenging; crop resistance may be overcome by new pathogen races while new pathogens have been introduced by global agricultural markets. Tomato is extensively used as a model plant for resistance studies and much has been attained through both genetic and biotechnological approaches. In this paper, we illustrate genomic methods currently employed to preserve resistant germplasm and to facilitate the study and transfer of resistance genes, and we describe the genomic organization of R-genes. Patterns of gene activation during disease resistance response, identified through functional approaches, are depicted. We also describe the opportunities offered by the use of new genomic technologies, including high-throughput DNA sequencing, large-scale expression data production and the comparative hybridization technique, whilst reporting multifaceted approaches to achieve genetic tomato disease control. Future strategies combining the huge amount of genomic and genetic data will be able to accelerate development of novel resistance varieties sustainably on a worldwide basis. Such strategies are discussed in the context of the latest insights obtained in this field.

## Introduction

Tomato (*Solanum lycopersicum*) is one of most important vegetable crops worldwide. This species is susceptible to over 200 diseases caused by all types of pathogens, including viruses, bacteria, fungi and nematodes (Lukyanenko 1991). Chemical control is often too expensive for growers and in some cases ineffective. Moreover, the use of such chemicals has been reduced due to environmental and consumer constraints. Hence understanding the basis of tomato–pathogen interactions and the development of resistant cultivars are important research goals for achieving sustainable agriculture.

Tomato health management can be particularly challenging due both to resistance being overcome by new pathogen races and to the introduction of new pathogens by global agricultural markets. To date, the most important gene family involved in pathogen recognition analyzed in tomato has been that of resistance genes (R-genes). R-genes encode proteins that recognize avirulent (Avr) pathogen proteins and initiate the defence mechanisms culminating in a hypersensitive response (HR). Plant immune systems can also respond to an infection through sensitization of their basal immune system that shares elements with the R-gene mediated response (Postel and Kemmerling 2009). Most commercial cultivars possess R-genes that confer resistance to fusarium wilt, verticillium wilt, root-knot nematode, alternaria stem canker, gray leaf spot, and some bacterial and viral diseases. For several tomato diseases such as early blight, powdery mildew, bacterial canker and bacterial wilt, horizontal resistance has been identified. For late blight and powdery mildew both vertical and horizontal resistances are available (Foolad 2007).

Tomato is extensively used as a model plant for resistance studies. Much has been achieved through the classical genetic approach (Ji et al. 2007). Current advances in plant biotechnology, including structural and functional genomics, can provide important tools for tomato improvement in developed and developing countries (Matsukura et al. 2008). During the last two decades, the use of molecular markers has facilitated identification, mapping and transfer of many disease resistance genes into tomato (Foolad 2007; Labate et al. 2007). A considerable number of studies have been undertaken to ascertain the molecular basis of resistance mechanisms underlying the defence process and plant–pathogen interactions. Numerous advances have been made in our knowledge of *Verticillium dahliae*, *Fusarium oxysporum, Cladosporium fulvum*, *Pseudomonas syringae pv. tomato,* tomato spotted wilt virus (TSWV), and tomato yellow leaf virus (TYLC) *Meloidogyne* spp. resistance processes, and steps toward the genetic control of these pathogens have also been taken (van Ooijen et al. 2007). In some areas where resistance genes or agronomic strategies are already used to control some serious diseases, others have emerged such as viruses (Hanssen et al. 2010) and *Tuta absoluta*, which can affect tomato crops (Desneux et al. 2010). It is therefore very important to implement a multifaceted approach toward disease control that is based both on a comprehensive knowledge of host–pathogen interactions and on a connected genomic strategy. In this way isolation of new tomato R-genes and their transfer through breeding approaches can bring many benefits in terms of ecology, economics and health for a growing sustainable agriculture.

This paper reports an overview of different biotechnology approaches available for improving tomato disease resistance. Methods employed to preserve resistant germplasm and explore structural genomic features are illustrated. We report recent advances to elucidate the role and mechanism of action of genes involved in the tomato defence response process. Opportunities offered by emerging technologies are discussed in the context of the latest insights obtained in this field. Future strategies that combine the huge amount of genetic and genomic information to facilitate the transfer of resistance genes are highlighted.

## Conservation and exploitation of genetic resources

Wild tomato species represent the primary source of resistance for tomato crops. Overall, resistances to over 40 major diseases have been discovered in tomato wild relatives, and at least 20 of them have been bred into tomato cultivars (Ji et al. 2007; Robertson and Labate 2007). *Solanum chilense*, *S.peruvianum*, *S .habrochaites* and *S. pimpinellifolium* have proved to be the richest source of resistance genes (Foolad and Sharma 2005; Laterrot 2000; Scott and Gardner 2007). Several resources and molecular approaches have been developed to fully exploit genetic potential in tomato breeding. Molecular markers have been used to characterize and conserve genetic resources (Ercolano et al. 2005; Nuez et al. 2004) for estimating genetic relationships (Albrecht et al. 2010; Spooner et al. 2005; Zuriaga et al. 2009) and managing Genebank accessions (Tanksley and McCouch 1997). Exotic libraries for analyzing tomato wild species diversity were obtained for several species (Eshed and Zamir 1995; Monforte and Tanksley 2000). A platform for developing and screening tomato introgression lines from different wild species was obtained in the framework of the EU-SOL project (Tripodi et al. 2010). In order to isolate resistance genes involved in disease response, several cDNA libraries and genomic libraries were developed from wild tomato species. For instance, Hemaprabha and Balasaraswathi (2009) recently built up a cDNA library from *S. peruvianum* EC52071 to perform screening for resistance genes against tospoviruses. Regrettably, most are scattered throughout individual laboratories and there is no centralized recording procedure. The SGN repository reported EST data of screening performed on *S. pennellii* and *S. habrochaites* cDNA libraries (http://solgenomics.net/search/search=library). The Texas A&M University genomic resources index reported the presence of a BAC library obtained from *S. pennellii* and *S.* *cheesmaniae* (http://hbz7.tamu.edu/homelinks/bac_est/bac.htm). Recently, oligonucleotide-based arrays have been used to identify DNA sequence polymorphisms in four different *S. pimpinellifolium* accessions for a study of polymorphism among *S. lycopersicum* and its closely related wild species (Sim et al. 2009). Rapidly increasing throughput will allow more species to be sequenced and more individuals to be genotyped at greater depth and hence with greater accuracy. We expect it to be possible to sequence tens of thousands of markers in thousands of individuals in the near future (Davey et al. 2011). Genome-wide genotyping using next generation sequencing could result in a very valuable bar coding method to be explored for future needs. Insight into these questions will greatly help estimate the wealth of resistance germplasm and enable tomato resources to be preserved and utilized efficiently.

## Structural analysis of R-loci

**Fig. 1:**
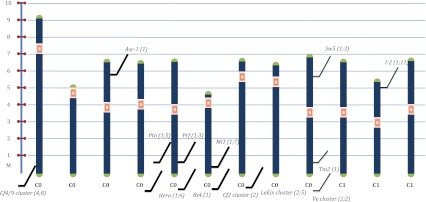
Tomato physical map with indication of cloned R-gene localization. The first number in brackets reports the number of functional genes, the second the number of genes in the resistance cluster

The tomato genome has been extensively explored with a view to elucidating the structure and organization of resistance loci. In particular, the availability of tomato molecular linkage maps has accelerated the process of disease gene localization. More than 100 loci underlying resistance traits have been mapped (Foolad 2007). By using molecular markers, chromosome walking and linkage analysis, several tomato R-genes were isolated, including *PTO*, *CF5*, *CF9*, *MI1*-*2*, *I2*, *ASC*, *HERO*, *VE*, *BS4* and *SW5* (Brandwagt et al. 2000; Dixon et al. 1998; Ernst et al. 2002; Kawchuk et al. 2001; Milligan et al. 1998; Ori et al. 1997; Parniske et al. 1997; Schornack et al. 2004). Thanks to the modular structure of plant R-genes it was possible to perform detailed structural analyses. This information was used to shed light on many sequences homologous to genes already isolated in the same species or related species and to isolate new resistance genes. The *TM2* gene was cloned by designing PCR primers on the *TM2*-*2* gene sequence obtained by a transposon tagging approach (Lanfermeijer et al. 2003), and many genes of *CF* series were isolated using the homology-based approach (Dixon et al. 1998; Parniske et al. 1997). Figure 1 reports the physical map based on recently released tomato genome sequences, of cloned resistance genes and of relative clusters in tomato.

Comparative approaches have revealed that resistance genes in Solanaceae are located in well-defined genomic regions (hot spots), which are organized in clusters and are conserved among related species (Ashrafi et al. 2009; Gebhardt and Valkonen 2001; Grube et al. 2000; Pan et al. 2000). Macrosynteny in the Solanaceae has been shown to be feasible. Each species has an array of R-genes targeting a given pathogen or pathogen family, and the subset of genes mapped thus far in different genera by chance is orthologous in related positions. The cloning of the late blight resistance gene *R3a* from potato based on *I2* in tomato illustrates the potential of these comparative approaches (Huang et al. 2005). In recent years, there has been a spurt of interest in the evolutionary dynamics of disease resistance in wild Solanaceae species (Hoekstra 2009; Rose et al. 2007; Wang et al. 2008). Identification of resistance gene homologues to determine genes involved in plant defence can enrich the repertoire of R-genes available for breeding purposes (Caicedo and Schaal 2004; Riely and Martin 2001). The technique capitalizes on the presence of conserved regions of resistance genes for designing primers and isolating resistance gene homologues from different plant genomes using the polymerase chain reaction (PCR) or more advanced sequencing techniques. Discovering the means of resistance loci arrangement will be crucial for generating novel or diverse pathogen recognition capabilities in order to overcome new disease challenges. The advent of second-generation sequencing enables the production of large quantities of genome sequence data at relatively low cost. This tool can greatly facilitate comparative genomics and gene discovery. Assessing R-loci variation in a wild population or in breeding resources will be a great challenge in tomato. Target-enrichment sequencing strategies, based on polymerase chain reaction (PCR) (Tewhey et al. 2009), hybridization or molecular inversion probes (Mamanova et al. 2010) are also available. The costs and time required to generate and map them are often not justified when only a specific region of the genome needs to be investigated and merely variations detected, without isolation of the intact allele. Moreover, the variations lying in highly duplicated and highly identical R-loci are still difficult to resolve. How R-genes varied and how many of these genes are conserved remains to be determined. Genomic information can be employed to link important disease resistance traits to sequence variations and incorporate this knowledge into crop improvement strategies. The interpretation of polymorphisms will require reliable methods to identify natural genetic variations, including combinations of variations, in a format suitable for downstream analysis.

## Dissection of R-gene mediated response

Many genes are activated during tomato disease resistance response, and several are specific to each plant–pathogen interaction. In the past decade, dissection of plant-defence mechanisms has led to the identification and isolation of numerous tomato defence players. To exert their function, PRF, I2 and BS4 proteins physically interact with the molecular chaperon complex composed by the heat shock protein 90 (HSP90), RAR1 and SGT1 (Bhaskar et al. 2008). A lipase-like protein (EDS1) was reported as being involved in their defence mechanism as well as in CF-4 and VE resistance responses (Hu et al. 2005). A domain-swap experiment conducted between MI-1.2 and MI-1.1 suggests that activation of NB-LRR proteins is likely to require a series of conformational changes, possibly mediated via nucleotide exchange/hydrolysis by the central nucleotide-binding site (Takken and Tameling 2009). Many RLP genes can physically interact with other proteins like CF-9-CITRX, LeEIX1-EDH2 and VE1-SERK3 (Fradin et al. 2009; Rivas et al. 2004). Interestingly, during the interaction between tomato and *Pseudomonas syringae* a series of proteins (PTO interacting proteins) were identified that play different roles in the various stages of defence response (Zhou et al. 1995). Ongoing genomic research will undoubtedly lead to further refinement of current models. Functional genomics could be very useful to investigate the features of plant–pathogen interactions. Various technologies have been developed to deduce and quantify the transcriptome, including hybridization or sequence-based approaches. Transcriptome comparison analysis has become a successful tool to gain valuable information on disease resistance response. Transcriptional changes in tomato plants during compatible and incompatible interactions with a range of pathogens were assessed (Table 1). Bhattarai et al. (2008) identified differences in JA pathway regulation in incompatible and compatible interactions with *Meloidogyne* spp., suggesting that the nematode is able to manipulate to its advantage by leveraging the existing cross talk between the JA and SA signalling pathways. Significant changes in expression of many unreported genes, involved in tomato–*Globodera rostochiensis* interaction, were detected through comparative serial analysis of gene expression (SAGE) and cDNA-AFLP (Uehara et al. 2007). Microarray technology was used to underline changes occurring in tolerant interaction of the fungal wilt pathogen *Verticillium dahliae* (Robb et al. 2007). Van Esse et al. (2009) evidenced that photorespiration, hypoxia and glyoxylate metabolism are induced upon infection of the vascular pathogen *Verticillium dahliae* and repressed during interaction with the foliar pathogen *Cladosporium fulvum*. Catoni et al. (2009) observed differences in the ABA metabolism in tomato root and shoot transcriptional response during Tomato Spotted Wilt Virus (TSWV) infection. On tracing the expression profile of Tomato–*Clavibacter michiganensis* subsp. *michiganensis* (Cmm) interaction, Balaji et al. (2008) evidenced that ethylene perception is involved in the regulation of Cmm-induced symptoms. Mysore et al. (2002), using GeneCalling, explored the tomato–*Pseudomonas syringae* interaction, evidencing that PRF protein acts very early on during the plant–pathogen interaction. Hanssen et al. (2011) showed perturbation of pigment biosynthesis during Pepino Mosaic virus infection. Functional approaches helped identify the dynamic changes involved in hormone regulation, plant pathogen defence response, cell cycle and cytoskeleton regulation, cell wall modification, cellular signalling, transcriptional regulation and primary metabolism. Regulation of typical defence protein families like chaperone MAP kinases and protein kinases, PR proteins, ubiquitin, oxidative burst-related proteins, transcription factors, and proteins involved in primary and secondary metabolism has been highlighted (Panthee and Chen 2010). The aims of transcriptomic analysis improved with the advent of RNA-Seq technology that allows the mapping of transcribed regions at a very high resolution. All species of transcripts, including mRNAs, non-coding RNAs and small RNAs can be catalogued; the transcriptional structure of genes, in terms of their start sites, 5′ and 3′ ends, splicing patterns and other post-transcriptional modifications can be determined; the change in expression levels of each transcript under different conditions can be quantified. Future investigation of gene regulation elements, such as epigenetic DNA modifications and the plethora of small non-coding RNAs, will be useful to better direct research. For instance, it was recently shown that microRNAs (miRNAs) participate in broad regulating R-gene expression on the post-transcriptional level, and play a vital role in the network of gene expression and regulation (Zhou et al. 2011). By computational prediction and experimental validation, most of the targets of miRNAs are transcription factors. Thereby the genes targeted by miRNAs control may be regulated by pathogen response (Luan et al. 2010). In particular, a substantial network of miRNAs and resulting phased small RNA (phasiRNAs) that target NB-LRR genes was identified in legumes and others species (Zhai et al. 2011). These data suggest that miRNAs result as master regulators of this large gene family via the targeting of highly conserved, protein-coding motifs. An extensive study for identifying and profiling the expression of miRNAs under various pathological conditions could better elucidate their specific role. Furthermore, many biological questions can only be addressed at the protein level as the presence of either a gene or its mRNA is no guarantee of a role in cellular activity. Large-scale proteome data sets are an important resource for the better understanding of protein functions in cellular systems. Proteomics has contributed to defining the specific functions of genes and proteins involved in plant–pathogen interactions. A group of molecular chaperones were identified in resistant plants challenged by bacteria (Coaker et al. 2004; Afroz et al. 2009; Dahal et al. 2010). Pr proteins in tomato plants challenged by *Fusarium oxysporum* (Houterman et al. 2007) and virus (Rodrigo et al. 1991) during interaction response were identified. However, technical limitations in proteomic studies need to be overcome in order to advance our knowledge on protein expression (Afroz et al. 2011). Over the last few years also the parallel assessment of the levels of a broad range of metabolites have been documented in tomato–pathogen interaction (López-Gresa et al. 2010). Direct chemical screening proved to be a powerful way to characterize genetic diversity in trichome-specialized metabolism (Schilmiller et al. 2010). The ability to screen a wide range of metabolites at once is very useful. Not only does this enable the detection of unknown traits but it also facilitates a greater understanding of the metabolic network and how this interacts with phenotypes (Fernie and Schauer 2009). In addition, large-scale collections of bioresources, such as mass-produced mutant lines and clones of full-length cDNAs and their integrative databases, could be useful for designing experiments (Aoki et al. 2010, Saito et al. 2011).

**Table 1:** Main transcriptomic tomato–pathogen interaction experiments undertaken

Taxonomic classification	Species	R-gene	Study	No. of differentially expressed genes	Biological function	Percentage of gene related to a biological function (%)	References
Nematode	Meloidogyne spp*.*	*Mi*	Microarray	1,941	Transcription	23	Bhattarai et al. (2008) Swiecicka et al. (2009)
					Defence-related	7	
					Stress response	4	
					Primary metabolism	10	
					Unknown	56	
	*Globodera rostochiensis*	*Hero*	SAGE	55	Transcription	1	Uehara et al. (2007)
					Unknown	5	
Fungi	*Cladosporium fulvum*	*Cf*	Microarray	7,073	Transcription	9	Van Esse et al. (2009)
					Stress response	8	
					Primary metabolism	58	
	*Verticillium dahliae*	*Ve*	Microarray	2,216	Transcription	6	Van Esse et al. (2009)
					Stress response	12	
					Primary metabolism	90	
Bacteria	*Clavibacter michiganensis*	*Cmm*	Microarray	161	Transcription	19	Balaji et al. (2008)
					Defence-related	36	
					Stress response	20	
					Primary metabolism	12	
					Secondary metabolism	3	
					Unknown	17	
	*Pseudomonas syringae*	*Pto*	Gene calling	432	Transcription	6	Mysore et al. (2002)
					Defence-related	10	
					Stress response	17	
					Primary metabolism	30	
					Secondary metabolism	8	
					Miscellaneous	4	
					Unknown	24	
Virus	*Tomato Spotted Wilt Virus*	*Sw*-*5*	Microarray	2,962	Defence and stress response	23	Catoni et al. (2009)
					Primary metabolism	46	
					Secondary metabolism	22	
					Signal transduction	9	

Information is reported about pathogens, involved R-genes, number of differentially expressed genes, functional annotations and test references

In the last few years, several research efforts have sought to give a comprehensive view of specific disease resistance responses in tomato. The amount of information about different aspects of the biology of this crop, as well as the many tools available for them and the number of scientists dedicated to their research creates a synergism that puts them at great advantage over other plant species. Current achievements in this research area have greatly advanced our understanding of tomato defence responses. A significant fraction of proteins identified through functional approaches lack functional information, highlighting the limitation in our current understanding of the defence process (Jones and Dang 2006). Characterization of the single genes is essential to provide biological insights and to further support established networks. Overexpression of tomato PTO proved to enhance expression of mitogen-activated protein kinases (MAPKs), conferring resistance against *Xanthomonas campestris* pv *vesicatoria* and *Cladosporium fulvum* (Tang et al. 1999). Tobacco N (Whitham et al. 1996), potato R1 (Faino et al. 2010) and pepper BS2 R-genes (Tai et al. 1999) showed they were specifically expressed also in tomato. Furthermore, the *OxO* gene (wheat oxalate oxidase) reduces light blight symptoms and improves *Botrytis cinerea* and *Sclerotinia sclerotiorum* resistance (Walz et al. 2008), and the sweet pepper ferredoxin-I protein *(PFLP)* improves resistance to *Ralstonia solanacearum* (Huang et al. 2007). Exploring mutant collections in order to develop Targeting-induced local lesions in genome (TILLING) platforms could represent a valuable high-throughput reverse genetic strategy to screen for point mutations in specific regions of targeted genes (Minoia et al. 2010). Furthermore, an effort to make knockout collections and silencing experiments could also be useful to identify unique features of each pathosystem. Techniques like artificial micro-RNA expression (Ruiz-Ferrer and Voinnet 2009; Ultzen et al. 1995), RNA interference (RNAi) (Bendahmane and Gronenborn 1997) and virus-induced gene silencing (VIGS) (Fu et al. 2005) could lend an impetus to basic plant–pathogen interaction studies and to improve plant–defence responses (Oh and Martin 2011).

## Emerging genomics tools

High-throughput sequencing and computational technologies have marked the beginning of a new genomics era. The genomic approach to exploring repertoires of resistance genes could clarify numerous molecular and evolutionary mechanisms for this gene family. Use of such technologies will make it easier to design diagnostic tests, conduct comparative and functional analysis and perform breeding by in silico design. DNA sequencing technologies are being updated at a blistering pace. These methodologies are transforming what we can do, how we should do it, and how much we can do in our own experiments. Because most platforms can be used for different applications, economics, length of time to acquire data, downstream analysis constraints become important for selecting a platform (Glenn 2011). As the number and variety of instruments increase and costs continue to decrease, we will become constrained only by our knowledge of the systems and our creativity to develop and adapt techniques to obtain data efficiently (Braeutigam and Gowik 2010).

Tomato represents one of best-explored model plants for studying defence response systems. Its genome sequence was recently released by the International SOL consortium using a Whole Genome Shotgun approach, including ~350,000 BAC and fosmid end-sequence pairs. The draft versions are accessible from the SOL Genomics Network (http://solgenomics.net/). New insights into the plant immune system can be achieved through genomic approaches. Starting from raw data it is possible to select a specific set of candidate genes putatively involved in biotic stress response. Expression levels of specific genes, differential splicing, and allele-specific expression of transcripts can be accurately determined by RNA-Seq experiments. All these attributes are not readily achievable via previously widespread hybridization-based or tag sequence-based approaches. However, the unprecedented level of sensitivity and the large amount of available data produced by NGS (next generation sequencing) platforms provide clear advantages as well as new challenges and issues.

Global information on tomato defence responses can create a body of knowledge concerning the frequency of relevant sequences, their evolution and possible functions. The development of tools to pool information obtained through different systems, to connect and to compare information in molecular biology and biochemistry, could be useful to start to delineate a systems biology approach in order to understand the plant-defence mechanism, thereby allowing new breeding methods to be designed. A combinatorial approach using multiple omics platforms and integration of their outcomes is now an effective strategy for clarifying molecular systems integral for plant improvement. Promotion of comparative genomics among model and applied plants allows us to grasp the biological properties of each species and to accelerate gene discovery and functional analyses of genes (Mochida and Shinozaki 2010). Interdisciplinary approaches can be undertaken using these resources for an in-depth study of the plant immune system. However, little attention has been given to integrating conceptually all of the related components identified in any plant–pathogen interaction. The most important point for a network construction is to obtain reliable analytical results based on sufficient experimental data. Signal transduction pathways should be connected and regulatory relationships between signals from elicitors and signal molecules need to be investigated. Several studies have been performed to draw out resistance gene features, analyze the level of conservation between organisms and to understand how they work. Collecting all the existing data in a repository could be a good starting point to conduct further studies. A specific online resource, the plant resistance gene (PRG) database was designed for molecular and in silico studies on plant R-genes (Sanseverino et al. 2010a). This manually curated database holds well characterized and candidate plant disease resistance genes belonging to nearly 200 plant species. Users can download reference genes of interest to design primers to amplify homologous genes in their species of interest or simply use various queries provided to get further information on domains, motifs and bibliography. Moreover, comparative studies and plant–pathogen interaction analysis can be performed through Pathoplant, that is, a database on plant–pathogen interactions and components of signal transduction pathways related to plant pathogenesis. Pathoplant also harbours gene expression data from *Arabidopsis thaliana* microarray experiments to enable the search for specific genes regulated upon pathogen infection or elicitor treatment (Bülow et al. 2009). The tomato functional genomics database also offers a valuable collection of tomato microarray experiments (Fei et al. 2011). The Dana Farber Cancer Institute Gene Index Database, also known as the “TIGR EST database” located at http://compbio.dfci.harvard.edu/tgi/tgipage.htm, the Mibase at http://www.kazusa.or.jp/jsol/microtom (Yano et al. 2006) and the TomatoEST db (D’agostino et al. 2007) can be used to manage and explore expressed sequences. ORTom, tomato-centred EST data-mining based on conserved co-expression, can be used to predict functional relationships among genes and to prioritize candidate genes for targeted studies (Miozzi et al. 2010). Moreover, the Solanaceae Genomic Network (SOL) offers several useful bioinformatics tools to make synteny studies (Mueller et al. 2005). It makes information available in an intuitive comparative format, thereby facilitating a systems approach to investigations into the basis of adaptation and phenotypic diversity.

The tomato reference genome is available and several tomato genomes have begun to be sequenced (SOL100 initiative; http://solgenomics.net/organism/sol100/view). A sequencing-based approach using these promising technologies could lead to identifying thousands of sequences of putative R-genes in a wide array of species. In principle, there are two possible approaches to discovering new genes based on genomic sequence and based on transcriptome sequences. A low coverage is required for the identification of genes and gene promoters. Reads of any length can be mapped onto the reference genome, and several algorithms for SNPs discovering have been developed. In addition, identification of related disease resistance genes from expressed gene messages (mRNA) would be compelling evidence for a potential function. Routine use of massively parallel sequencing will require higher accuracy, better ways to select genomic subsets of interest, and improvements in processing speed. Selection of accurate SNP sites, e.g. with high-quality value and/or with high coverage of sequence fragments, is also important (Shirasawa et al. 2010). High-throughput genotyping and phenotyping projects of large populations require sophisticated laboratory information management systems. In order to obtain valuable information, data need to be handled with care.

The ability to screen a wide range of metabolites at once will also be very useful. Several recent studies have illustrated the utility of combining data from metabolomics with those from other genomics platforms to provide new insights on both gene annotation (Mintz-Oron et al. 2008) and regulation in complex biological systems (Osorio et al. 2011; Klee 2010). These approaches have resulted in the identification of numerous candidate genes. The aim of these non-targeted ‘omic’ technologies is to extend our understanding beyond the analysis of separate parts of the system, in contrast to traditional reductionist hypothesis-driven approaches. The integration of genotyping, pheno/morphotyping and the analysis of the molecular phenotype using metabolomics, proteomics and transcriptomics will reveal a novel understanding of plant genome and its interaction with the environment. Core facilities handling cooperative projects will require a straightforward solution to manage combined information. Due to these great advances in technologies it now seems to be the perfect time to exploit genome information to make new achievements in this field.

## Advancements in breeding strategy design

**Fig. 2:**
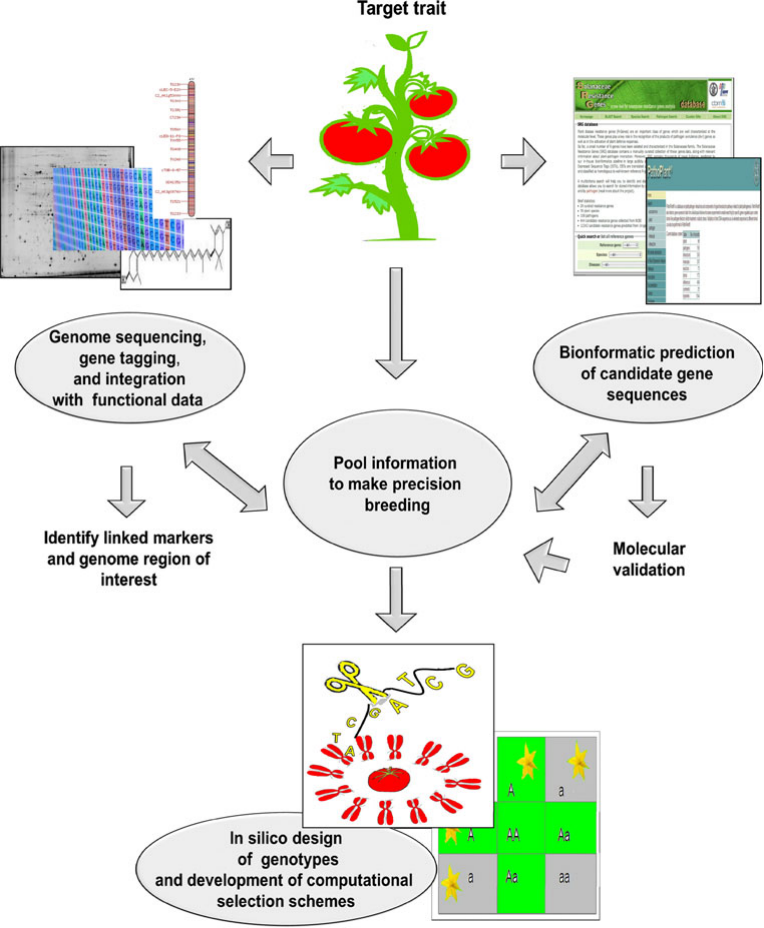
A schematic view of combined genomic strategies to obtain tomato resistance cultivars

The necessary reliance on resistance processes to work out the genetic basis of variation for resistance traits is not limiting, given the extensive work conducted in solanaceae species. DNA markers tightly linked to resistance loci have long been used for marker-assisted selection (MAS) to incorporate these valuable traits in new tomato varieties. They help carry a more efficient and precise transfer of the R-gene/QTL, reducing the negative effects of linkage drag (St Clair 2010). MAS tomato selection has also proved to accelerate the pyramiding of desirable genes and QTLs for different traits (Barone and Frusciante 2007). To increase selection efficiency, an approach that combines the use of high-throughput genomic analysis with phenotypic analysis could help identify candidate genes for genomics-assisted breeding (Fig. 2).

A high-precision breeding approach can be achieved using a tomato physical map that allows specific traits to be detected. Merging literature data, genetic information and prediction data is an efficient way to trace tomato R-genes (Sanseverino et al. 2010b). So-called ‘‘jackpot’’ cultivars can be seen as a source of cassettes of resistances and contain clusters of many tightly-linked resistances (Grube et al. 2000). The targeted genome region can be in silico selected, well characterized by molecular work and transferred during whole genome selection (WGS). To coordinate high-density SNP genotyping of varieties and lines and organize precise phenotyping efforts for association studies a comprehensive tomato diversity survey can be very useful (Robbins et al. 2011). Allele-specific markers should be informative whatever the genetic background, but sometimes the presence of very similar R-gene paralogues could invalidate diagnostic testing as many resistance genes remain in (large) clusters, containing highly similar gene members. The complex arrangement of the disease-resistance loci *I2, MI* and *SW*-*5* genes in tomato (Dianese et al. 2010; El Mohtar et al. 2007; Simons et al. 1998) has shown that design of specific primers can be very difficult. The availability of linked markers, allele-specific markers and sequence data sets can facilitate the screening of varieties and populations for many resistance genes at the same time.

The acceleration in mapping and sequencing techniques and the decreasing in costs in NGS and metabolomics-based phenotyping, will extend the possibilities of gene and marker discovery and genome-wide quantification of gene expression. Integrating results from metabolic and morphological profiling proves to be a powerful strategy for crop improvement. Most metabolomics approaches are unbiased; the profiles they produce contain many unannotated peaks, representing unknown metabolites. Therefore, it seems likely that the power of metabolomics as a platform for the selection of breeding material can only improve. The measurement of the dynamic molecular phenotype should be connected to the static genotype information. Based on the integration of genotype data, especially in conjunction with SNP measurements, a systematic investigation of this intimate relationship is possible by means of dynamic transcriptomic, proteomic and metabolomic data. Recently, a systematic approach was proposed explicitly on the basis of a genotype–phenotype–equation (Weckwerth 2011).

Importance of the genetic background should not be underestimated. Gene dosage effects, non-allelic and epistatic interactions, and host background genotypic factors could influence the inheritance patterns of R-genes and also affect the phenotypes they mediate. Detailed analysis of parental lines can help to define the molecular, biochemical and phenotypic components of disease response. A comprehensive understanding of the process will translate into knowledge-based approaches in genome-assisted breeding approaches. A current challenge in interpreting genome-wide association studies is to establish the mechanistic links between the measured genotype and observed phenotype (Tian et al. 2011). This information provides an opportunity for determining reliability of using different ‘omic’ profiling techniques. In silico procedures are expected to improve the breeding strategies, especially when the numbers of genotypes and traits to assess are huge. After generating and analyzing new populations, information from informatics support could help understand and interpret the resulting data. The genetic advance achieved through genomic selection depends on the ability to capture superior alleles, the repeatability of the trait and the selection pressure imposed. Parental line selection in breeding hybrid varieties is an important task. An important criterion for the choice of parents is their genetic distance. The relatedness of parents can be researched by comparing their genome. Those parents with a polymorphism in target traits should then be crossed.

Modern breeding is a dynamic and evolving research discipline. Traditional selection schemes should be modified and adapted for computational methods. Algorithms that generate both general and detailed scores of each trait for each genotype should be developed for handling large data sets and methods for estimating recombination rates, and recombination hotspots should be identified. The individuals can be sorted with respect to their general scores to extract resistant genotypes with the desired traits. Germplasm should then be selected based on the likelihood the lines will produce valuable new genetic combinations.

## Conclusions

Exploring the genetic and genomic basis of genomic variation can be useful for identifying new resistance genes and clarifying their mechanisms of action. Enormous advances have been made in our knowledge of R-genes and in elucidating the role and mechanism of action of genes involved in the tomato defence response. New genomic technologies, including high–throughput DNA sequencing, large-scale expression data production and comparative hybridization techniques have led to the expansion of available data. Methods for identifying modification events and interactions in the plant proteome, and for measuring the abundance of many metabolites simultaneously are also available. The overall reduction in costs has led to experiments being designed in which R-gene investigation will prove more productive. Such comprehensive biological vision provides an excellent starting point for designing experiments, generating hypotheses or conceptualization of model based on integrated knowledge. In this context, extensive information for different purposes is available in database repositories and constitutes a valuable set of data for studies, characterization and use of resistance genes in breeding programs.
